# Recent developments on the role of epigenetics in obesity and metabolic disease

**DOI:** 10.1186/s13148-015-0101-5

**Published:** 2015-07-11

**Authors:** Susan J. van Dijk, Ross L. Tellam, Janna L. Morrison, Beverly S. Muhlhausler, Peter L. Molloy

**Affiliations:** CSIRO Food and Nutrition Flagship, PO Box 52, North Ryde, NSW 1670 Australia; CSIRO Agriculture Flagship, 306 Carmody Road, St Lucia, QLD 4067 Australia; Early Origins of Adult Health Research Group, School of Pharmacy and Medical Sciences, Sansom Institute for Health Research, University of South Australia, GPO Box 2471, Adelaide, SA 5001 Australia; FOODplus Research Centre, Waite Campus, The University of Adelaide, PMB 1, Glen Osmond, SA 5064 Australia; Women’s and Children’s Health Research Institute, 72 King William Road, North Adelaide, SA 5006 Australia

**Keywords:** Epigenetics, DNA methylation, Obesity, Type 2 diabetes, Developmental programming

## Abstract

The increased prevalence of obesity and related comorbidities is a major public health problem. While genetic factors undoubtedly play a role in determining individual susceptibility to weight gain and obesity, the identified genetic variants only explain part of the variation. This has led to growing interest in understanding the potential role of epigenetics as a mediator of gene-environment interactions underlying the development of obesity and its associated comorbidities. Initial evidence in support of a role of epigenetics in obesity and type 2 diabetes mellitus (T2DM) was mainly provided by animal studies, which reported epigenetic changes in key metabolically important tissues following high-fat feeding and epigenetic differences between lean and obese animals and by human studies which showed epigenetic changes in obesity and T2DM candidate genes in obese/diabetic individuals*.* More recently, advances in epigenetic methodologies and the reduced cost of epigenome-wide association studies (EWAS) have led to a rapid expansion of studies in human populations. These studies have also reported epigenetic differences between obese/T2DM adults and healthy controls and epigenetic changes in association with nutritional, weight loss, and exercise interventions. There is also increasing evidence from both human and animal studies that the relationship between perinatal nutritional exposures and later risk of obesity and T2DM may be mediated by epigenetic changes in the offspring. The aim of this review is to summarize the most recent developments in this rapidly moving field, with a particular focus on human EWAS and studies investigating the impact of nutritional and lifestyle factors (both pre- and postnatal) on the epigenome and their relationship to metabolic health outcomes. The difficulties in distinguishing consequence from causality in these studies and the critical role of animal models for testing causal relationships and providing insight into underlying mechanisms are also addressed. In summary, the area of epigenetics and metabolic health has seen rapid developments in a short space of time. While the outcomes to date are promising, studies are ongoing, and the next decade promises to be a time of productive research into the complex interactions between the genome, epigenome, and environment as they relate to metabolic disease.

## Introduction

Obesity is a complex, multifactorial disease, and better understanding of the mechanisms underlying the interactions between lifestyle, environment, and genetics is critical for developing effective strategies for prevention and treatment [1].

In a society where energy-dense food is plentiful and the need for physical activity is low, there is a wide variation in individuals’ susceptibility to develop obesity and metabolic health problems. Estimates of the role of heredity in this variation are in the range of 40–70 %, and while large genome-wide association studies (GWAS) have identified a number of genetic loci associated with obesity risk, the ~100 most common genetic variants only account for a few percent of variance in obesity [2, 3]. Genome-wide estimates are higher, accounting for ~20 % of the variation [3]; however, a large portion of the heritability remains unexplained.

Recently, attention has turned to investigating the role of epigenetic changes in the etiology of obesity. It has been argued that the epigenome may represent the mechanistic link between genetic variants and environmental factors in determining obesity risk and could help explain the “missing heritability.” The first human epigenetic studies were small and only investigated a limited number of loci. While this generally resulted in poor reproducibility, some of these early findings, for instance the relationship between *PGC1A* methylation and type 2 diabetes mellitus (T2DM) [4] and others as discussed in van Dijk et al. [5], have been replicated in later studies. Recent advances and increased affordability of high-throughput technologies now allow for large-scale epigenome-wide association studies (EWAS) and integration of different layers of genomic information to explore the complex interactions between the genotype, epigenome, transcriptome, and the environment [6–9]. These studies are still in their infancy, but the results thus far have shown promise in helping to explain the variation in obesity susceptibility.

There is increasing evidence that obesity has developmental origins, as exposure to a suboptimal nutrient supply before birth or in early infancy is associated with an increased risk of obesity and metabolic disease in later life [10–13]. Initially, animal studies demonstrated that a range of early life nutritional exposures, especially those experienced early in gestation, could induce epigenetic changes in key metabolic tissues of the offspring that persisted after birth and result in permanent alterations in gene function [13–17]. Evidence is emerging to support the existence of the same mechanism in humans. This has led to a search for epigenetic marks present early in life that predict later risk of metabolic disease, and studies to determine whether epigenetic programming of metabolic disease could be prevented or reversed in later life.

This review provides an update of our previous systematic review of studies on epigenetics and obesity in humans [5]. Our previous review showcased the promising outcomes of initial studies, including the first potential epigenetic marks for obesity that could be detected at birth (e.g*.*, *RXRA*) [18]. However, it also highlighted the limited reproducibility of the findings and the lack of larger scale longitudinal investigations. The current review focuses on recent developments in this rapidly moving field and, in particular, on human EWAS and studies investigating the impact of (pre- and postnatal) nutritional and lifestyle factors on the epigenome and the emerging role of epigenetics in the pathology of obesity. We also address the difficulties in identifying causality in these studies and the importance of animal models in providing insight into mechanisms.

## Review

### Epigenetic changes in animal models of obesity

Animal models provide unique opportunities for highly controlled studies that provide mechanistic insight into the role of specific epigenetic marks, both as indicators of current metabolic status and as predictors of the future risk of obesity and metabolic disease. A particularly important aspect of animal studies is that they allow for the assessment of epigenetic changes within target tissues, including the liver and hypothalamus, which is much more difficult in humans. Moreover, the ability to harvest large quantities of fresh tissue makes it possible to assess multiple chromatin marks as well as DNA methylation. Some of these epigenetic modifications either alone or in combination may be responsive to environmental programming. In animal models, it is also possible to study multiple generations of offspring and thus enable differentiation between transgenerational and intergenerational transmission of obesity risk mediated by epigenetic memory of parental nutritional status, which cannot be easily distinguished in human studies. We use the former term for meiotic transmission of risk in the absence of continued exposure while the latter primarily entails direct transmission of risk through metabolic reprogramming of the fetus or gametes.

Animal studies have played a critical role in our current understanding of the role of epigenetics in the developmental origins of obesity and T2DM. Both increased and decreased maternal nutrition during pregnancy have been associated with increased fat deposition in offspring of most mammalian species studied to date (reviewed in [11, 13–15, 19]). Maternal nutrition during pregnancy not only has potential for direct effects on the fetus, it also may directly impact the developing oocytes of female fetuses and primordial germ cells of male fetuses and therefore could impact both the offspring and grand-offspring. Hence, multigenerational data are usually required to differentiate between maternal intergenerational and transgenerational transmission mechanisms.

Table 1 summarizes a variety of animal models that have been used to provide evidence of metabolic and epigenetic changes in offspring associated with the parental plane of nutrition. It also contains information pertaining to studies identifying altered epigenetic marks in adult individuals who undergo direct nutritional challenges. The table is structured by suggested risk transmission type.

**Table 1:** Evidence for a role of epigenetics in animal models of obesity separated by transmission type

Transmission type	Species	Experimental model	Phenotype affected	Epigenetic changes	Ref.
Intergenerational maternal effect	Sheep	Periconceptional undernutrition in normal and overweight ewes using artificial insemination and embryo transfer	Fat deposition and adrenal changes in offspring	Decreased expression of *IGF2* and decreased DNA methylation of a proximal imprinting control region; changes in adrenal *IGF2* DNA methylation; hypermethylation of pituitary glucocorticoid receptor	[14, 88–91]
Intergenerational maternal effect	Sheep	Maternal undernutrition prior to conception and during early gestation	Programming of obesity	Altered offspring histone methylation and acetylation in fetal hypothalamic energy regulating pathways	[20]
Intergenerational maternal effect	Sheep	Different maternal dietary energy sources during that last half of gestation	Late gestation fetal gene expression and DNA methylation from a variety of tissues	Changes in late gestation fetal DNA methylation of CpG islands associated with *IGF2R* and *H19* in muscle and adipose tissue	[23]
Intergenerational maternal effect	Pig	Methylating micronutrient supplementation during gestation—impacts on F2	Back fat percentage, adipose tissue, and fat thickness at 10th rib, croup, and shoulder in F2	Differentially expressed metabolic genes in F2 liver and muscle, DNA methylation change in *IYD*	[31]
Intergenerational maternal effect	Mouse	Maternal low-protein diet during gestation and maternal diet restriction during gestation	Body weight, food intake, and adiposity	Altered germline DNA methylation of F1 adult males in a locus specific manner; changed expression and DNA methylation of *LXRA* in liver; demethylation of leptin promoter in adipocytes	[22, 26, 92]
Intergenerational maternal effect	Mouse	Maternal high-fat diet during gestation; maternal obesity model; maternal high-fat diet using a Glut4^+/−^ genetic background; maternal diet-induced obesity	Offspring chromatin organization; metabolic syndrome in offspring unmasked by exposure to western diet; glucose intolerance, insulin resistance, hepatic steatosis; obesity; exacerbated metabolic syndrome in offspring; insulin levels, insulin resistance in adipose tissue	Changes in offspring hepatic histone marks H3K14ac and H3K9me3; changes in offspring hepatic gene expression and widespread subtle changes in cytosine methylation; DNA methylation change in *PEG3* in spermatozoa of offspring; cell autonomous transmission of altered insulin signaling. Reduced *IRS1* expression associated with elevated miR126	[21, 24, 28, 93]
Intergenerational maternal effect	Rat	Maternal diet restriction during gestation; suboptimal diet during early gestation	Catch up growth, obesity, and liver weight; T2D	Change in offspring liver *IGF1* expression and *IGF1* H3K4 methylation; decreased *PPARA* expression and increased DNA methylation in the *PPARA* promoter in liver; dhange in offspring growth hormone and *PPARA* expression and DNA methylation in liver; chromatin changes affecting enhancer/promoter interactions at *HNF4A* promoter in pancreatic islets from offspring	[17, 25, 29, 30]
Intergenerational maternal effect	Rat	Maternal overfeeding model during preconception and gestation	Adipogenesis, gene expression and reduced representation DNA methylation in offspring	Changes in gene expression and proximal DNA methylation in genes in lipogenic pathways of adipocytes from offspring	[16]
Intergenerational maternal effect	Macaque	Maternal high-fat diet during gestation	Altered expression of *Npas2*	Changes in offspring fetal liver chromatin mark H3K14ac in the *NPAS2* promoter	[27]
Intergenerational paternal effect	Drosophila	Paternal overnutrition	Obesity in offspring	Chromatin (H3K9me3 and H3K27me3)-dependent reprogramming of offspring metabolic genes; a similar system may regulate obesity susceptibility and phenotypic variation in mice and humans	[33]
Intergenerational paternal effect	Mouse	Paternal low-protein diet	High cholesterol in offspring	Changes in hepatic gene expression and DNA methylation in offspring	[32]
Intergenerational paternal effect	Mouse	Intrauterine growth restriction	F1 offspring become obese and glucose intolerant with aging	F1 males show change in methylation of *LXRA* in sperm that is transmitted to somatic cells in the F2	[34]
Intergenerational paternal effect	Mouse	Paternal prediabetes	F1 has increased susceptibility to diabetes	F1 show changes in pancreatic gene expression and DNA methylation linked to insulin signaling. A large portion of these genes are also differentially methylated in sperm	[35]
Potential transgenerational effect	Mouse	A^vy^ mouse—change in coat color and adult onset obesity through maternal transmission to the next generation; modulation by methyl donors and genistein during gestation.	Coat color and adult onset obesity in offspring	DNA methylation of a retrotransposon promoter adjacent to the *agouti* gene; evidence for germ line transmission of methylation status	[38–41, 94]
Effect resulting from direct exposure of adult	Mouse	High-fat diet	Weight, fasting glucose, glucose, and insulin tolerance tests; obesity	Differential DNA methylation at numerous sites in adipose tissue; changes in DNA methylation of metabolism-related genes in liver and oocytes	[43, 95]

*T2D* type 2 diabetes

(i) Epigenetic changes in offspring associated with maternal nutrition during gestation

Maternal nutritional supplementation, undernutrition, and overnutrition during pregnancy can alter fat deposition and energy homeostasis in offspring [11, 13–15, 19]. Associated with these effects in the offspring are changes in DNA methylation, histone post-translational modifications, and gene expression for several target genes, especially genes regulating fatty acid metabolism and insulin signaling [16, 17, 20–30]. The diversity of animal models used in these studies and the common metabolic pathways impacted suggest an evolutionarily conserved adaptive response mediated by epigenetic modification. However, few of the specific identified genes and epigenetic changes have been cross-validated in related studies, and large-scale genome-wide investigations have typically not been applied. A major hindrance to comparison of these studies is the different developmental windows subjected to nutritional challenge, which may cause considerably different outcomes. Proof that the epigenetic changes are causal rather than being associated with offspring phenotypic changes is also required. This will necessitate the identification of a parental nutritionally induced epigenetic “memory” response that precedes development of the altered phenotype in offspring.

(ii) Effects of paternal nutrition on offspring epigenetic marks

Emerging studies have demonstrated that paternal plane of nutrition can impact offspring fat deposition and epigenetic marks [31–34]. One recent investigation using mice has demonstrated that paternal prediabetes leads to increased susceptibility to diabetes in F1 offspring with associated changes in pancreatic gene expression and DNA methylation linked to insulin signaling [35]. Importantly, there was an overlap of these epigenetic changes in pancreatic islets and sperm suggesting germ line inheritance. However, most of these studies, although intriguing in their implications, are limited in the genomic scale of investigation and frequently show weak and somewhat transient epigenetic alterations associated with mild metabolic phenotypes in offspring.

(iii) Potential transgenerational epigenetic changes promoting fat deposition in offspring

Stable transmission of epigenetic information across multiple generations is well described in plant systems and *C. elegans*, but its significance in mammals is still much debated [36, 37]. An epigenetic basis for grandparental transmission of phenotypes in response to dietary exposures has been well established, including in livestock species [31]. The most influential studies demonstrating effects of epigenetic transmission impacting offspring phenotype have used the example of the viable yellow agouti (A^vy^) mouse [38]. In this mouse, an insertion of a retrotransposon upstream of the *agouti* gene causes its constitutive expression and consequent yellow coat color and adult onset obesity. Maternal transmission through the germ line results in DNA methylation mediated silencing of *agouti* expression resulting in wild-type coat color and lean phenotype of the offspring [39, 40]. Importantly, subsequent studies in these mice demonstrated that maternal exposure to methyl donors causes a shift in coat color [41]. One study has reported transmission of a phenotype to the F3 generation and alterations in expression of large number of genes in response to protein restriction in F0 [42]; however, alterations in expression were highly variable and a direct link to epigenetic changes was not identified in this system.

(iv) Direct exposure of individuals to excess nutrition in postnatal life

While many studies have identified diet-associated epigenetic changes in animal models using candidate site-specific regions, there have been few genome-wide analyses undertaken. A recent study focussed on determining the direct epigenetic impact of high-fat diets/diet-induced obesity in adult mice using genome-wide gene expression and DNA methylation analyses [43]. This study identified 232 differentially methylated regions (DMRs) in adipocytes from control and high-fat fed mice. Importantly, the corresponding human regions for the murine DMRs were also differentially methylated in adipose tissue from a population of obese and lean humans, thereby highlighting the remarkable evolutionary conservation of these regions. This result emphasizes the likely importance of the identified DMRs in regulating energy homeostasis in mammals.

### Human studies

Drawing on the evidence from animal studies and with the increasing availability of affordable tools for genome-wide analysis, there has been a rapid expansion of epigenome studies in humans. These studies have mostly focused on the identification of site-specific differences in DNA methylation that are associated with metabolic phenotypes.

**Fig. 1:**
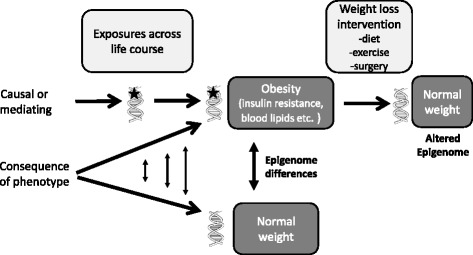
Epigenetic changes as a cause or consequence of obesity and related comorbidities. An epigenetic change is indicated as a *star* on the DNA

A key question is the extent to which epigenetic modifications contribute to the development of the metabolic phenotype, rather than simply being a consequence of it (Fig. 1). Epigenetic programming could contribute to obesity development, as well as playing a role in consequent risk of cardiovascular and metabolic problems. In human studies, it is difficult to prove causality [44], but inferences can be made from a number of lines of evidence:

(i) Genetic association studies. Genetic polymorphisms that are associated with an increased risk of developing particular conditions are a priori linked to the causative genes. The presence of differential methylation in such regions infers functional relevance of these epigenetic changes in controlling expression of the proximal gene(s). There are strong cis-acting genetic effects underpinning much epigenetic variation [7, 45], and in population-based studies, methods that use genetic surrogates to infer a causal or mediating role of epigenome differences have been applied [7, 46–48]. The use of familial genetic information can also lead to the identification of potentially causative candidate regions showing phenotype-related differential methylation [49].
(ii) Timing of epigenetic changes. The presence of an epigenetic mark prior to development of a phenotype is an essential feature associated with causality. Conversely, the presence of a mark in association with obesity, but not before its development, can be used to exclude causality but would not exclude a possible role in subsequent obesity-related pathology.
(iii) Plausible inference of mechanism. This refers to epigenetic changes that are associated with altered expression of genes with an established role in regulating the phenotype of interest. One such example is the association of methylation at two CpG sites at the *CPT1A* gene with circulating triglyceride levels [50]. *CPT1A* encodes carnitine palmitoyltransferase 1A, an enzyme with a central role in fatty acid metabolism, and this is strongly indicative that differential methylation of this gene may be causally related to the alterations in plasma triglyceride concentrations.

#### Epigenome-wide association studies: identifying epigenetic biomarkers of metabolic health

A number of recent investigations have focused on exploring associations between obesity/metabolic diseases and DNA methylation across the genome (Table 2). The largest published EWAS so far, including a total of 5465 individuals, identified 37 methylation sites in blood that were associated with body mass index (BMI), including sites in *CPT1A*, *ABCG1*, and *SREBF1* [51]. Another large-scale study showed consistent associations between BMI and methylation in *HIF3A* in whole blood and adipose tissue [52], a finding which was also partially replicated in other studies [9, 51]. Other recently reported associations between obesity-related measures and DNA methylation include (i) DNA methylation differences between lean and obese individuals in *LY86* in blood leukocytes [53]; (ii) associations between *PGC1A* promoter methylation in whole blood of children and adiposity 5 years later [54]; (iii) associations between waist-hip ratio and *ADRB3* methylation in blood [55]; and (iv) associations between BMI, body fat distribution measures, and multiple DNA methylation sites in adipose tissue [9, 56]. EWAS have also shown associations between DNA methylation sites and blood lipids [55, 57–59], serum metabolites [60], insulin resistance [9, 61], and T2DM [48, 62, 63] (Table 2).

**Table 2:** Representative human studies showing evidence for a role of epigenetics in obesity and related comorbidities

	Phenotype	Association with epigenetic marks or changes in epigenetic marks^a^	Includes validation^b^	Ref.
Cross-sectional studies				
BMI, WC		Methylation in 37 CpGs associated with BMI and 1 probe with WC in blood (*n* = 5465); of those 16 CpGs (e.g., CpGs in *CPT1A*, *ABCG1*, and *SREBPF1*) were also associated with BMI in subcutaneous adipose tissue (*n* = 648)	Yes, 3 other cohorts	[51]
BMI		DNA methylation of 4979 CpGs (e.g., in *FTO*, *TCF7L2*, *FASN*, *PGC1A*, *CCRL2*) in subcutaneous adipose tissue (*n* = 190). Several BMI-related methylation sites were also associated with age (e.g., *ELOVL2*), and with gene expression levels	Yes, 2nd cohort	[9]
BMI		*HIF3A* methylation 3 CpGs in whole blood (*n* = 2587) and subcutaneous adipose tissue (*n* = 635), not in skin (*n* = 395)	Yes, 3 other cohorts	[52]
Obesity		*LY86* methylation 1 CpG in blood leukocytes adolescents and adults (*n* = 1534)	Yes, 4 other cohorts	[53]
Obesity		249 DMRs in subcutaneous adipose tissue (*n* = 21), methylation in all these 249 regions also changed with high-fat feeding in mice and includes regions overlapping T2DM loci such as in *TCF7L2*	No, only in mice	[43]
WHR, blood pressure LDL cholesterol		Association between WHR and *ADRB3* methylation in whole blood and between blood pressure and *ADRB3* methylation in visceral adipose tissue of obese (*n* = 25). Association between *ADRB3* methylation and LDL cholesterol in whole blood of men with familial hypercholesterolemia (*n* = 41)	Not validated in 2nd cohort	[55]
BMI		*ADCY3* meQTL in subcutaneous adipose tissue from twins (*n* = 648)	Yes, technical	[8]
BMI		Methylation differences at 1236 CpGs in leukocytes of monozygotic twins discordant for BMI and liver fat (*n* = 13)	Yes, technical	[96]
Adiposity phenotypes		Methylation in 101 genes in subcutaneous adipose tissue (*n* = 106) including *AOC3*, *SOD3*, *DOCK9*, *AQP7*, *ANGPT4*, *ANGPT2*, *TIMP4*, *ADAMST4*, *HOXA3*, and *LIPE*, not found in blood leukocytes same individuals	No validation performed	[56]
TG, VLDL		*CPT1A* methylation 1 CpG in CD4+T cells (*n* = 991) and leukocytes (*n* = 1261)	Yes, technical and 2nd cohort	[57]
VLDL and LDL		*CPT1A* methylation 2 CpGs in CD4+T cells (*n* = 994)	Yes, in the same cohort	[58]
Cholesterol and TG		Methylation in 9 CpGs, including in *ABCG1, CPT1A* and *SREBF1*, in whole blood (*n* = 2747), 5 of these CpGs also showed associations in subcutaneous adipose tissue (*n* = 634)	Yes, 2 other cohorts	[59]
Insulin and HOMA-IR		*ABCG1* 1 CpG in CD4+T cells (*n* = 837)	Yes, in the same cohort	[61]
HbA1C		DNA methylation of 711 CpGs in subcutaneous adipose tissue (*n* = 96 males). Not validated in female cohort (*n* = 94) but 30 CpGs validated in T2DM case-control cohort	Yes, validated in 1 of 2 cohorts	[9]
T2DM		*MALT1* methylation whole blood (*n* = 27 twins + *n* = 263 individuals) and other less significant DMRs (FDR < 0.1) overlapping T2DM GWAS loci	Yes, other cohort	[48]
T2DM		Methylation in 1649 CpG sites, some overlapping T2DM, and obesity GWAS loci such as *TCF7L2*, *FTO*, and *KCNQ1* in pancreatic islets (*n* = 49)	Yes, technical	[62]
T2DM		No differentially methylated sites (after FDR correction) in T2DM discordant monozygotic twins (*n* = 14 pairs). Differential methylation at 15,627 CpGs, including in T2DM GWAS loci *PPARG*, *KCNQ1*, *TCF7L2*, and *IRS1* in subcutaneous adipose tissue (*n* = 56). 1410 of these CpGs were also differentially methylated (*P* < 0.05) in the T2DM discordant twins	Yes, other cohort	[63]
Longitudinal studies				
Adiposity measured annually age 9–14 years		Increase *PGC1A* promoter methylation in whole blood children measured annually from 5–7 years (*n* = 40)	No, but measures at multiple time points	[54]
Maternal exposure or phenotype and epigenetic marks in offspring				
Prenatal famine		181 DMRs in adult whole blood (*n* = 48), including in *CDH23*, *SMAD7*, *INSR*, *CPT1A*, *KLF13*, *RFTN1* (validated in *n* = 120)	Yes, technical and 2nd cohort	[68]
Variation methyl donor intake		Changes in mean methylation across *BOLA3*, *LOC654433*, *EXD3*, *ZFYVE28*, *RBM46*, and *ZNF678* in blood leukocytes (*n* = 126) and hair follicles (*n* = 82) of 2- to 8-month infants	No validation performed	[69]
Periconceptional BMI		Decreased mean methylation across *BOLA3*, *LOC654433*, *EXD3*, *ZFYVE28*, *RBM46*, and *ZNF678* in blood leukocytes (*n* = 126) and hair follicles (*n* = 82) of 2- to 8-month infants	No validation performed	[69]
Gestational weight gain early pregnancy		Increased methylation 4 CpGs in *MMP7*, *KCNK4*, *TRPM5*, and *NFKB1* in newborn cord blood (*n* = 88), no association in 2nd cohort (*n* = 170)	Not validated (technical and 2nd cohort)	[71]
Preconceptional BMI		Differential methylation in *ZCCHC10* in newborn cord blood (*n* = 308), other less significant sites were found in *WNT16*, *ACPL2*, *C18orf8*, *ANGPTL2*, *SAPCD2*, and *ADCY3*	No	[73]
Gestational Diabetes		42 CpGs in newborn cord blood (*n* = 136). ~1/3 of CpGs overlapped with sites that were associated with maternal glucose levels (*n* = 36) or micronutrient supplementation (*n* = 59) in 2 other child cohorts	Yes, technical and 2 other cohorts	[70]
Gestational Diabetes		No differentially methylated sites (after FDR correction) in cord blood and placenta (*n* = 44) but enrichment of sites in genes metabolic disease pathway	No	[72]
Intervention				
Weight loss surgery		Change in methylation at 3601 CpGs (195 DMRs) in subcutaneous adipose tissue and 15 CpGs in omental adipose tissue (*n* = 15), some DMRs overlapping known obesity and T2DM loci	Yes, technical	[64]
Weight loss surgery		227 DMRs, methylation in these regions also changed with high-fat feeding in mice	No, only in mice	[43]
Weight loss surgery in liver disease		Before surgery 467 differentially methylated CpGs between control (*n* = 18), healthy obese (*n* = 18), steatosis (*n* = 12), and NASH (*n* = 15) liver samples. After surgery changes in methylation at 113 CpGs, disease-associated methylation was reversible at the *HOXB1*, *PRKCZ*, *SLC38A10*, and *SECTM1* loci	No, but for baseline technical and 2nd cohort	[79]
Weight loss		Methylation profiles of *RYR1, TUBA3C* and *BDNF* in PBMCs of successful weight loss maintainers (*n* = 16) more closely resembled lean (*n* = 16) than obese subjects (*n* = 16)	No	[77]
Endurance and strength exercise		Changes in DNA methylation in skeletal muscle of obese T2DM subjects (*n* = 17) after 16 weeks, most pronounced with endurance exercise	No	[83]
High-fat diet (5 days)		*PPARGC1A* DNA methylation across 4 CpGs increased in subcutaneous adipose tissue of lean adults born with low birth weight (*n* = 19) but not in controls (*n* = 26)	No	[76]

*BMI* body mass index, *DMR* differentially methylated region, *GDM* gestational diabetes mellitus, *GWAS* genome-wide association study, *NASH* non-alcoholic steatohepatitis, *PBMC* peripheral blood mononuclear cell, *T2DM* type 2 diabetes mellitus, *TG* triglycerides, *WC* waist circumference, *WHR* waist-hip ratio

^a^If not otherwise stated the number of subjects includes the total number in discovery and validation sets

^b^Validation refers to validation of at least some epigenetic marks identified in the primary study cohort. This can be either technical validation, using another method for the measurement of epigenetic marks, or validation in another cohort

From these studies, altered methylation of *PGC1A*, *HIF3A*, *ABCG1*, and *CPT1A* and the previously described *RXRA* [18] have emerged as biomarkers associated with, or perhaps predictive of, metabolic health that are also plausible candidates for a role in development of metabolic disease.

#### Interaction between genotype and the epigenome

Epigenetic variation is highly influenced by the underlying genetic variation, with genotype estimated to explain ~20–40 % of the variation [6, 8]. Recently, a number of studies have begun to integrate methylome and genotype data to identify methylation quantitative trait loci (meQTL) associated with disease phenotypes. For instance, in adipose tissue, an meQTL overlapping with a BMI genetic risk locus has been identified in an enhancer element upstream of *ADCY3* [8]. Other studies have also identified overlaps between known obesity and T2DM risk loci and DMRs associated with obesity and T2DM [43, 48, 62]. Methylation of a number of such DMRs was also modulated by high-fat feeding in mice [43] and weight loss in humans [64]. These results identify an intriguing link between genetic variations linked with disease susceptibility and their association with regions of the genome that undergo epigenetic modifications in response to nutritional challenges, implying a causal relationship. The close connection between genetic and epigenetic variation may signify their essential roles in generating individual variation [65, 66]. However, while these findings suggest that DNA methylation may be a mediator of genetic effects, it is also important to consider that both genetic and epigenetic processes could act independently on the same genes. Twin studies [8, 63, 67] can provide important insights and indicate that inter-individual differences in levels of DNA methylation arise predominantly from non-shared environment and stochastic influences, minimally from shared environmental effects, but also with a significant impact of genetic variation.

#### The impact of the prenatal and postnatal environment on the epigenome

##### Prenatal environment

Two recently published studies made use of human populations that experienced “natural” variations in nutrient supply to study the impact of maternal nutrition before or during pregnancy on DNA methylation in the offspring [68, 69]. The first study used a Gambian mother-child cohort to show that both seasonal variations in maternal methyl donor intake during pregnancy and maternal pre-pregnancy BMI were associated with altered methylation in the infants [69]. The second study utilized adult offspring from the Dutch Hunger Winter cohort to investigate the effect of prenatal exposure to an acute period of severe maternal undernutrition on DNA methylation of genes involved in growth and metabolism in adulthood [68]. The results highlighted the importance of the timing of the exposure in its impact on the epigenome, since significant epigenetic effects were only identified in individuals exposed to famine during early gestation. Importantly, the epigenetic changes occurred in conjunction with increased BMI; however, it was not possible to establish in this study whether these changes were present earlier in life or a consequence of the higher BMI.

Other recent studies have provided evidence that prenatal overnutrition and an obese or diabetic maternal environment are also associated with DNA methylation changes in genes related to embryonic development, growth, and metabolic disease in the offspring [70–73]. While human data are scarce, there are indications that paternal obesity can lead to altered methylation of imprinted genes in the newborn [74], an effect thought to be mediated via epigenetic changes acquired during spermatogenesis.

##### Postnatal environment

The epigenome is established de novo during embryonic development, and therefore, the prenatal environment most likely has the most significant impact on the epigenome. However, it is now clear that changes do occur in the “mature” epigenome under the influence of a range of conditions, including aging, exposure to toxins, and dietary alterations. For example, changes in DNA methylation in numerous genes in skeletal muscle and *PGC1A* in adipose tissue have been demonstrated in response to a high-fat diet [75, 76]. Interventions to lose body fat mass have also been associated with changes in DNA methylation. Studies have reported that the DNA methylation profiles of adipose tissue [43, 64], peripheral blood mononuclear cells [77], and muscle tissue [78] in formerly obese patients become more similar to the profiles of lean subjects following weight loss. Weight loss surgery also partially reversed non-alcoholic fatty liver disease-associated methylation changes in liver [79] and in another study led to hypomethylation of multiple obesity candidate genes, with more pronounced effects in subcutaneous compared to omental (visceral) fat [64]. Accumulating evidence suggests that exercise interventions can also influence DNA methylation. Most of these studies have been conducted in lean individuals [80–82], but one exercise study in obese T2DM subjects also demonstrated changes in DNA methylation, including in genes involved in fatty acid and glucose transport [83]. Epigenetic changes also occur with aging, and recent data suggest a role of obesity in augmenting them [9, 84, 85]. Obesity accelerated the epigenetic age of liver tissue, but in contrast to the findings described above, this effect was not reversible after weight loss [84].

Collectively, the evidence in support of the capacity to modulate the epigenome in adults suggests that there may be the potential to intervene in postnatal life to modulate or reverse adverse epigenetic programming.

#### Effect sizes and differences between tissue types

DNA methylation changes associated with obesity or induced by diet or lifestyle interventions and weight loss are generally modest (<15 %), although this varies depending on the phenotype and tissue studied. For instance, changes greater than 20 % have been reported in adipose tissue after weight loss [64] and associations between *HIF3A* methylation and BMI in adipose tissue were more pronounced than in blood [52].

The biological relevance of relatively small methylation changes has been questioned. However, in tissues consisting of a mixture of cell types, a small change in DNA methylation may actually reflect a significant change in a specific cell fraction. Integration of epigenome data with transcriptome and other epigenetic data, such as histone modifications, is important, since small DNA methylation changes might reflect larger changes in chromatin structure and could be associated with broader changes in gene expression. The genomic context should also be considered; small changes within a regulatory element such as a promotor, enhancer, or insulator may have functional significance. In this regard, DMRs for obesity, as well as regions affected by prenatal famine exposure and meQTL for metabolic trait loci have been observed to overlap enhancer elements [8, 43, 68]. There is evidence that DNA methylation in famine-associated regions could indeed affect enhancer activity [68], supporting a role of nutrition-induced methylation changes in gene regulation.

**Fig. 2:**
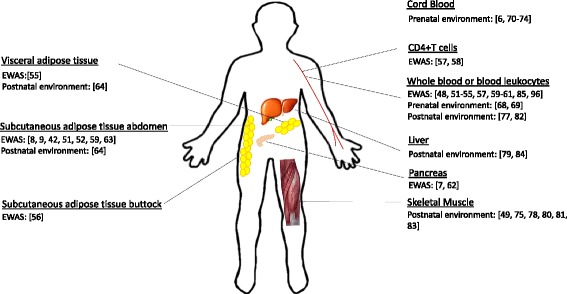
Overview of human tissues used for studies into the role of epigenetics in obesity. For each tissue, studies are grouped by study type; epigenome-wide association studies (EWAS) and pre- and postnatal interventions. *Numbers* represent the reference number

A major limitation in many human studies is that epigenetic marks are often assessed in peripheral blood, rather than in metabolically relevant tissues (Fig. 2). The heterogeneity of blood is an issue, since different cell populations have distinct epigenetic signatures, but algorithms have been developed to estimate the cellular composition to overcome this problem [86]. Perhaps more importantly, epigenetic marks in blood cells may not necessarily report the status of the tissues of primary interest. Despite this, recent studies have provided clear evidence of a relationship between epigenetic marks in blood cells and BMI. In the case of *HIF3A* for which the level of methylation (beta-value) in the study population ranged from 0.14–0.52, a 10 % increase in methylation was associated with a BMI increase of 7.8 % [52]. Likewise, a 10 % difference in *PGC1A* methylation may predict up to 12 % difference in fat mass [54].

## Conclusions

The study of the role of epigenetics in obesity and metabolic disease has expanded rapidly in recent years, and evidence is accumulating of a link between epigenetic modifications and metabolic health outcomes in humans. Potential epigenetic biomarkers associated with obesity and metabolic health have also emerged from recent studies. The validation of epigenetic marks in multiple cohorts, the fact that several marks are found in genes with a plausible function in obesity and T2DM development, as well as the overlap of epigenetic marks with known obesity and T2DM genetic loci strengthens the evidence that these associations are real. Causality has so far been difficult to establish; however, regardless of whether the associations are causal, the identified epigenetic marks may still be relevant as biomarkers for obesity and metabolic disease risk.

Effect sizes in easily accessible tissues such as blood are small but do seem reproducible despite variation in ethnicity, tissue type, and analysis methods [51]. Also, even small DNA methylation changes may have biological significance. An integrative “omics” approach will be crucial in further unraveling the complex interactions between the epigenome, transcriptome, genome, and metabolic health. Longitudinal studies, ideally spanning multiple generations, are essential to establishing causal relationships. We can expect more such studies in the future, but this will take time.

While animal studies continue to demonstrate an effect of early life nutritional exposure on the epigenome and metabolic health of the offspring, human data are still limited. However, recent studies have provided clear evidence that exposure to suboptimal nutrition during specific periods of prenatal development is associated with methylation changes in the offspring and therefore have the potential to influence adult phenotype. Animal studies will be important to verify human findings in a more controlled setting, help determine whether the identified methylation changes have any impact on metabolic health, and unravel the mechanisms underlying this intergenerational/transgenerational epigenetic regulation. The identification of causal mechanisms underlying metabolic memory responses, the mode of transmission of the phenotypic effects into successive generations, the degree of impact and stability of the transmitted trait, and the identification of an overarching and unifying evolutionary context also remain important questions to be addressed. The latter is often encapsulated by the predictive adaptive response hypothesis, i.e., a response to a future anticipated environment that increases fitness of the population. However, this hypothesis has increasingly been questioned as there is limited evidence for increased fitness later in life [87].

In summary, outcomes are promising, as the epigenetic changes are linked with adult metabolic health and they act as a mediator between altered prenatal nutrition and subsequent increased risk of poor metabolic health outcomes. New epigenetic marks have been identified that are associated with measures of metabolic health. Integration of different layers of genomic information has added further support to causal relationships, and there have been further studies showing effects of pre- and postnatal environment on the epigenome and health. While many important questions remain, recent methodological advances have enabled the types of large-scale population-based studies that will be required to address the knowledge gaps. The next decade promises to be a period of major activity in this important research area.
